# Fluorescence-Based Multiplex Protein Detection Using Optically Encoded Microbeads

**DOI:** 10.3390/molecules17032474

**Published:** 2012-03-01

**Authors:** Bong-Hyun Jun, Homan Kang, Yoon-Sik Lee, Dae Hong Jeong

**Affiliations:** 1 School of Chemical and Biological Engineering, Seoul National University, Seoul 151-747, Korea; Email: yslee@snu.ac.kr; 2 Nano Systems Institute and Interdisciplinary Program in Nano-Science and Technology, Seoul National University, Seoul 151-747, Korea; 3 Department of Chemistry Education, Seoul National University, Seoul 151-747, Korea

**Keywords:** optically-encoded bead, fluorescence, quantum dots, surface-enhanced Raman scattering (SERS), bead-based assay, label-free detection, high-throughput screening

## Abstract

Potential utilization of proteins for early detection and diagnosis of various diseases has drawn considerable interest in the development of protein-based multiplex detection techniques. Among the various techniques for high-throughput protein screening, optically-encoded beads combined with fluorescence-based target monitoring have great advantages over the planar array-based multiplexing assays. This review discusses recent developments of analytical methods of screening protein molecules on microbead-based platforms. These include various strategies such as barcoded microbeads, molecular beacon-based techniques, and surface-enhanced Raman scattering-based techniques. Their applications for label-free protein detection are also addressed. Especially, the optically-encoded beads such as multilayer fluorescence beads and SERS-encoded beads are successful for generating a large number of coding.

## 1. Introduction

High-throughput screening (HTS) of biomarkers has great potential for clinical and genetic analysis, and medical diagnostics. Because proteins can indicate the state of disease progression and the functions of normal biological processes within the human body, HTS techniques that identify proteins and their expression levels are very important for early detection, diagnosis, and therapy [1,2,3,4,5,6,7].

The most widely used method for protein analysis in basic research and clinical diagnostics is the enzyme-linked immunosorbent assay (ELISA) [8,9,10]. Mass spectrometry also plays a major role in protein analysis [11,12,13,14]. However, because these assay methods can only be used to analyze one or a few samples at a time, they are not suitable for high-throughput assays with reduced assay volumes [15,16].

Planar microarrays (protein chips) and bead-based microarrays (suspension arrays) are widely used to date for multiplex protein detection. The microarray chip-based screening has many advantages over ELISA such as assay miniaturization, multiplexing, low consumption of samples (less than a nanoliter), and high-throughput screening [17,18,19,20]. Thus, this method is now becoming one of the most powerful tools for multiplexed protein analysis. However, proteins can be expressed in a wide range (~10^6^ fold), and hence a large dynamic range of detection level is recommended for protein detection. In this regard, small sample volume on microarray spots may reduce the dynamic range of detection in some cases [21,22,23].

As one of widely used methods for the multiplex detection of biomolecules, bar-coded (encoded) micro-sized beads (microbeads) have been used in bead-based arrays (suspension or liquid arrays) [22,24,25]. These techniques have several advantages over the chip-based substrates in development of HTS systems for protein detection [15,26,27,28,29]: (1) beads can have larger surface areas than planar chips as illustrated by Luminex^TM^ claiming ~10^6^ capture molecules per bead. This means that more capture biomolecules can be immobilized on the bead, and thus bead-based arrays are more probable to detect a wide range of target proteins; (2) detection is faster and sensitivity is equal to or higher than that of ELISAs because the interaction between beads and target molecules can be nearly comparable with solution-phase kinetics; (3) target molecules can be collected by using flow cytometry such as fluorescence-activated cell sorting (FACS); (4) Large-scale fabrication and surface modification is possible, and the prepared beads can be stored. Thus, customization is possible by selective mixing of antibody-conjugated microbeads; (5) beads can be used with combination of microfluidic devices to detect trace amounts of molecules in a manner of automation. 

**Figure 1:**
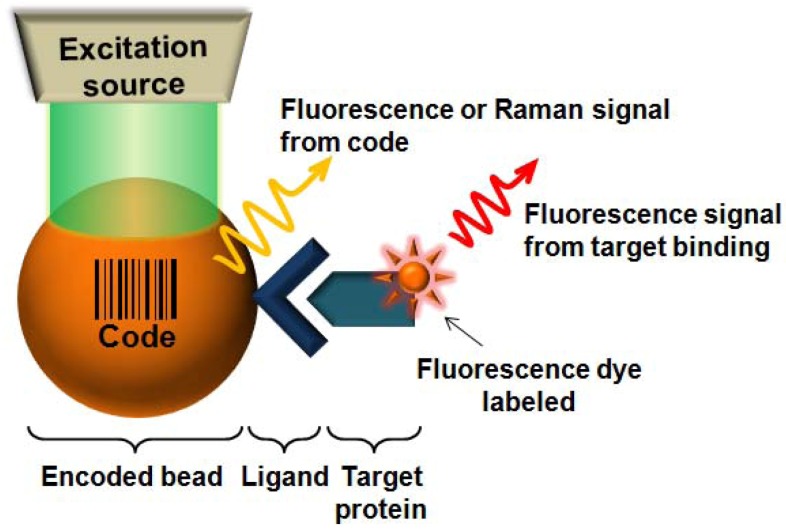
Schematic illustration of principle of a bead based assay.

In the microbead system, capture molecules that specifically bind to target analytes are immobilized to corresponding unique bar-coded micro-sized beads. By decoding the beads, the identity of captured analytes can be determined. Thus, the system needs two readouts as shown in Figure 1: (1) the bar-coded micro-sized beads for multiplexing; (2) the target binding events on each particle [30].

Until now, a number of encoding strategies such as chemical encoding, electronic encoding, graphical encoding and spectrometric encoding have been proposed and demonstrated [30,31,32]. Among various methods, optically encoded beads have been widely used with well developed optical readout tools [33,34,35], since decoding of optically encoded beads is non-invasive, and coding is stable in surface modification and protein binding [1,36,37,38].

There are various approaches for coding and decoding of beads, and for identification of the binding event of a protein with a capture molecule on beads. Fluorescence-based detection has been widely used to detect binding event owing to several major advantages: easy visualization, quantification of target molecules, selective excitation of fluorophores (for example, fluorescein isothiocyanate (FITC), rhodamine isothiocyanate (RITC), Cy series dyes or Alexa Fluor dyes) and fast readout [39,40].

Recently, our group has introduced several kinds of optically-encoded bead and together with strategies for protein binding event to solve some of the biggest challenges with bead-based arrays such as generating a large number of coding and label-free protein detection. This review is focused on fluorescence-based multiplex detection systems of proteins with particular emphasis on optically-encoded beads.

## 2. Fluorescence-Encoded Beads for Protein Detection

### 2.1. Fluorescence-Encoded Beads

Among the optical encoding methods, fluorescence-encoding has been most widely used in biological applications owing to the simple encoding process, easy detection of large-scale samples, and compatibility with a variety of biological chemistries [28,36,41]. The fluorescence-encoded beads are prepared by entrapping fluorescent dyes into microbeads composed of, for example, polystyrene. Microbeads can have various kinds of encoding by changing different dyes and controlling their concentrations.

The commercially available Luminex protein detection system is a representative example of fluorescence-encoded bead-based assays [22,42]. In the Luminex 100/200 system, polystyrene-based microbeads of 5.6-µm size (xMAP microspheres) are used as carriers and stained with precise proportions of red and orange fluorophores which denote the bead identity. The red or orange fluorophores in the microbeads are measured by photoexcitation with a red-colored laser light for decoding the identity of the target. The green dye on the microbeads is measured by photoexcitation with a green-colored laser light for quantification of the target protein. The green fluorescence intensity reflects the amount of targets since the fluorescence comes from the secondary probes added after target capture to form sandwich immunoassayor hybridization like ELISA. Moreover, the beads can be separated by both their optical properties and target amounts by combining with FACS. A new system (FLEXMAP 3D by Luminex) using three colors has been introduced to the market allowing to multiplex up to a degree of 500 identities.

Many researchers and several companies have developed microbead-based assay systems based on the Luminex system [1]. Very recently, non-commercial activities by the Lund-Johansen group resulted in a multiplex of 1725 beads. They combined SEC (size exclusion chromatography) to MAP (microsphere-based affinity proteomics) for measuring of large numbers of proteins simultaneously [43]. However, broad and overlapping feature of emission bands, complex optical system requiring multiple excitation lines, and the limit of practically available dyes hinder broad utilization of this method [32,44]. Several emission-based beads were developed to overcome these problems. One is quantum dot (QD)-embedded particles [45,46,47,48,49,50]. QDs, which are colloidal II–VI semiconductor nanocrystals with tunable fluorescence emission depending on their size, can overcome many problems of organic fluorescence-based beads. Their advantages include excitation in a broad range, narrow (20–30 nm) emission spectrum, photostability, high quantum yield of luminescence (20 times brighter), and good chemical stability. A large number of codings can be created by embedding QDs of different color into beads at precisely-controlled ratios of composition [45]. Theoretically, 10,000–40,000 different types of coding beads can be created by using several QD colors and six intensity levels. So far, various techniques for embedding QDs into microspheres have been reported and the prepared QD-embedded beads were used for multiplex assays [46,48,49]. Thus, QD-encoded beads have great potential to become one of the widely used types of optically-encoded beads.

The other approach to overcome the limited number of fluorescence-encoded beads is the use of localized fluorescence-encoded beads. One of the examples is fluorescence dye-doped NP-embedded bead [27,51,52,53,54] and the microparticles with dyes incorporated layer by layer. Although these approaches cannot beat QD-embedded beads in coding capacity, localization of fluorescence can increase the encoding capacity and has potential advantages over QD-based ones since fabrication and application of QDs can be limited several practical problem such as price of QDs, difficulty of a large quantity production, toxicity and hydrophobic properties.

**Figure 2:**
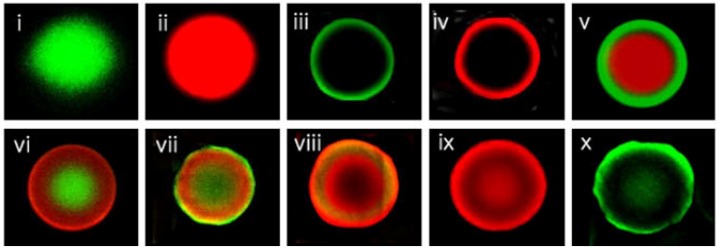
Fluorescence images of layer-by-layer fluorescence dye particles (reproduced with permission from reference [35]. Copyright 2010, Elsevier B.V.).

Recently, our group has developed and used layer-by-layer (multilayer) fluorescence-encoded beads for protein detection as illustrated in Figure 2 [35]. This system is produced by using several fluorescent dyes such as FITC and rhodamine through diffusion control of an Fmoc-protecting group into TentaGel resins. To control the diffusion rate of the Fmoc-protecting group, TentaGel amino resin is swollen in an aqueous HCl solution, and then Fmoc-OSu in organic solvent is added to protect some parts of the amino groups from the shell surface. Then, the rest of the amino groups are encoded with FITC or rhodamine. With repetitions of this process, 10 types of multi-layered fluorescence can be prepared with only two dyes. Biotin and a RNA-aptamer, which specifically recognize streptavidin and HCV helicase, respectively, are introduced to the multilayer fluorescence-encoded beads and monitored for their binding activities to the target molecules. GST-FITC target proteins are selectively bound to GST antibody-immobilized beads in a mixture of various ligand-immobilized beads. After binding of target (streptavidin-FITC, HCV helicase-Cy3, GST-FITC), the ligands are easily identified by their color codes.

### 2.2. Label-Free Protein Detection Using Optically-Encoded Beads

In order to apply the bead-encoding system to bio-detections, an additional labeling step is generally required to monitor protein-binding event. Moreover, the number of matched-pair antibodies in sandwich immunoassays is limited for multiplex detection.

**Figure 3:**
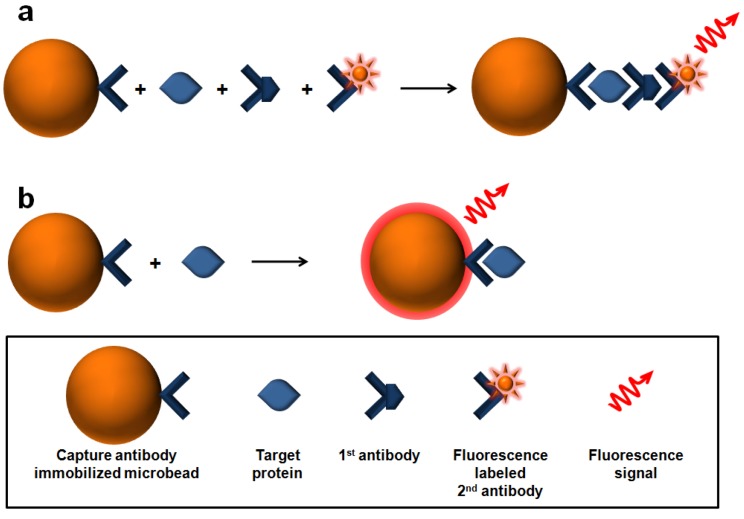
Schematic diagram of two types of the target binding recognition. (**a**) labeling method; (**b**) label free method.

Direct labeling method which is conjugating of fluorophores (e.g., Cy-3, Cy-5) to target can be used (Figure 3a). However, several key disadvantages such as generally lower signal intensity and flexibility, higher cost, complex labeling procedure limit their usefulness of direct labeling method [55,56].

Label-free techniques that monitors inherent property changes of the capture molecule by target binding can be used to avoid the above-mentioned problems, and many research groups are currently developing label-free planar chip assays based on various tools such as surface plasmon resonance (SPR), carbon nanotubes (CNTs), nanowires, nanohole arrays and interferometry [41,57]. The combination of label-free protein detection with beads within fluidic platform has been reported. Zhao *et al*. have introduced label-free analyses on inverse-opaline photonic beads [58]. In their system, target amount can be used for detection in monitoring the reflection-peak shift. This system has the potential to be combined with a microfluidic system. Our group has studied label-free protein detection using dielectrophoresis (DEP) force using a microfluidic system. Although this DEP-based approach for bead separation by target binding has potential, the current sensitivity is not enough for use in protein detection [59].

Recently, we combined aptamer-based molecular beacons (MBs) [37] or polydiacetylene (PDA) [60] with optically-beads, which can be used with a bead-based array system. The system is designed to generate fluorescence by binding event as shown in Figure 3b, and offers the additional advantage of separation by target protein amount using flow cytometry.

Using a sandwich immunoassay format, up to a maximum of 30 targets can be analyzed in multiplex within the same sample, which is circumvented by the other method such as direct labeling method or label-free detection. 

#### 2.2.1. Molecular Beacon-Based Protein Detection Methods

MBs have a hairpin structure that can undergo spontaneous conformational changes upon hybridization to complementary nucleic acids or protein targets, activating fluorescence resonance energy transfer (FRET) [61,62]. For example, the dye molecule does not emit lights when it is near a quencher, and it emits when it is distant from quencher.

MBs are attached to beads by electrostatic or biotin-streptavidin interactions to detect unlabeled nucleic acids in solution for multiplex detection [63]. Using beads of different sizes and MBs in two fluorophore colors, synthetic nucleic acid sequences were successfully recognized for three respiratory pathogens, including the SARS coronavirus in proof-of-concept experiments. Considering that routine flow cytometry can detect only up to four fluorescent channels, this assay approach may allow multiplex detection of nucleic acids in a single tube. However, there are several obstacles to overcome: For example, unstable interactions, random attachment of MBs, and the bulkiness of streptavidin.

**Figure 4:**
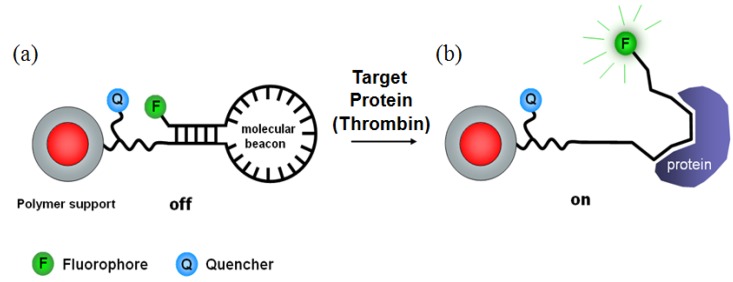
Schematic illustration of MBs on optically-encoded beads for detecting thrombin without additional labeling. (**a**) before thrombin addition; (**b**) after thrombin addition [37].

As a different approach, MBs were directly coupled to multilayer fluorescence-encoded beads by covalent bonding [37]. In this study, a RNA aptamer was used for thrombin detection and the MB was designed as a hairpin structure. One side of the RNA aptamer had a conjugated with a fluorescent dye, and the other side was immobilized to the beads containing quencher. By immobilization of these “apta-beacons” onto optically-encoded beads, core-shell type beads contain a fluorescent dye-encoded core and apta-beacon-coupled shell. In a model study, thrombin (100 nmol) was directly detected using this apta-beacon bead method. As illustrated in Figure 4, the fluorophore of the MB would be separated from the quencher to allow fluorescence emission (488 nm) when the MBs on the beads bind thrombin. Before thrombin treatment, the beads showed only red color (543 nm) from the rhodamine encoded at the core layer. The thrombin-bound apta-beacon beads were easily recognized by the appearance of fluorescence without any further labeling step. However, only several RNA aptamers have been reported for protein targeting. Because the known RNA aptamer sequence for targeting protein can be used for this method, applicable proteins can be highly limited in current stage.

#### 2.2.2. Polydiacetylene-Coated Coding Beads

PDA-based biosensors have attracted considerable attention due to their unique color change from blue to red in response to a variety of stimuli such as applied stress, changes in temperature or pH, and ligand-receptor binding. Thus, PDA-based biosensors have been applied to a wide range of analytes, including proteins, viruses, antibacterial peptides, antibodies, and pharmacologically active compounds.

**Figure 5:**
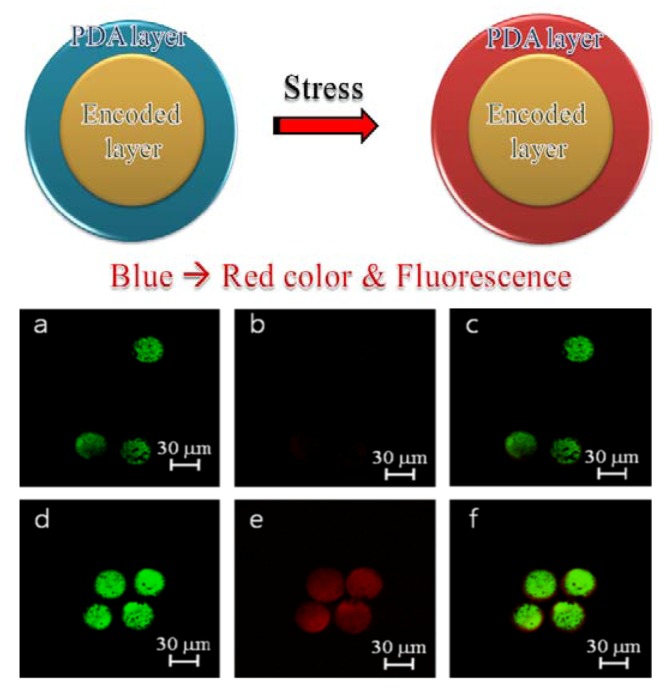
PDA-coated encoded beads. Illustration of PDA-coated encoded beads of before and after stress (upper panel), CLSM images of PDA–FITC encoded beads (lower panel) (**a**–**c**) unstressed beads and (**d**–**f**) stressed beads. (**a**,**d**) at a wavelength of 488 nm; (**b**,**e**) beads at a wavelength of 543 nm; (**c**,**f**) at wavelengths of 488 and 543 nm (reproduced with permission from reference [60]. Copyright 2011, Elsevier B.V.).

Most PDA-based biosensors are prepared in the form of free-floating vesicles of 100–200 nm or planar chips [64,65,66]. PDA-based biosensors can be combined with fluorescence-encoded materials for multiplex detection [60]. In order to combine the PDA to optically-encoded beads, core–shell type beads having an optically-coded core are prepared by adapting the preparation method of multilayer fluorescence-encoded beads. PDA is then coated onto the optically-encoded beads in a manner similar to the chip-based immobilization method, in which monomers are immobilized onto the substrate and then PDA is further coated onto it. The prepared PDA-coated beads provide encoding capability as well as the PDA sensing of a fluorescence signal and color change induced by external stress (Figure 5). Moreover several ligands and their immobilization methods, such as PDA monomer with biotin or alkyne group for click reactions [67], have been reported for PDA functionalization. However, because PDA property can be changed not only by antibody-antigen binding but also by the other stresses such as pH, and temperature, practical applications for high-throughput screening of target proteins can be sometimes limited.

Although these studies are at an early stage, the combination of optically-encoded beads with fluorescence-based methods could evolve as a powerful label-free detection method in such fields as separation using direct detection of ligand-target binding events, flow cytometry, multiplexing ability, and easy and real-time recognition of ligand type and binding event by using CLSM.

## 3. SERS-Encoded Beads for Protein Detection

Nanostructures of noble metal such as gold and silver exhibit an optical phenomenon known as surface-enhanced Raman scattering (SERS), which enhances Raman scattering of molecules adsorbed thereon. When SERS is used as a coding method, it has advantages for bioassays over other optical tools: (1) A large number of different Raman signatures can be obtained using different reporter molecules. Since SERS peaks are narrow (less than 0.5 nm), spectral overlap is minimized, and thus a large number of coding can be created by the combination of chemicals; (2) Choice of photoexcitation line is very flexible covering UV to NIR region; (3) There is no photobleaching in Raman scattering; (4) They can afford non-invasive analysis of biomolecules and thus are applicable to high-throughput screening of various biomolecules [68,69,70,71,72].

So far, a large number of SERS-coded materials and readout techniques have been reported [73,74,75,76,77,78,79]. Because mono-disperse size and homogeneous surface morphology of coding materials are important in suspension-array technology for comparison of protein loading levels, monodisperse-sized beads with SERS-codes have been manufactured for multiplex protein detection [34,38].

Monodisperse micro-sized polystyrene beads prepared by seed polymerization were used as stable templates for SERS encoding by our group. Silver NPs were embedded on sulfonated micro-beads polystyrene (PS) beads and then Raman-labeled organic compounds were adsorbed on the silver NPs. Then, the beads were coated with a silica shell using tetraethoxyorthosilicate (TEOS) for easy surface modification and chemical stability. The SERS-encoded beads had uniform size and produced highly intense and reproducible Raman signatures. Moreover, the size of PS beads could be controlled by changing backbone size, and additional function such as magnetic property can be incorporated to the SERS-encoded beads. The protein p53 which is tumor suppressor protein was chosen as a model to show that the SERS-encoded beads could be used for protein detection. By using p53 antibody-conjugated SERS-encoded beads, the p53 tumor suppressor protein in a protein mixture was successfully detected by sandwich-type bioassays.

**Figure 6:**
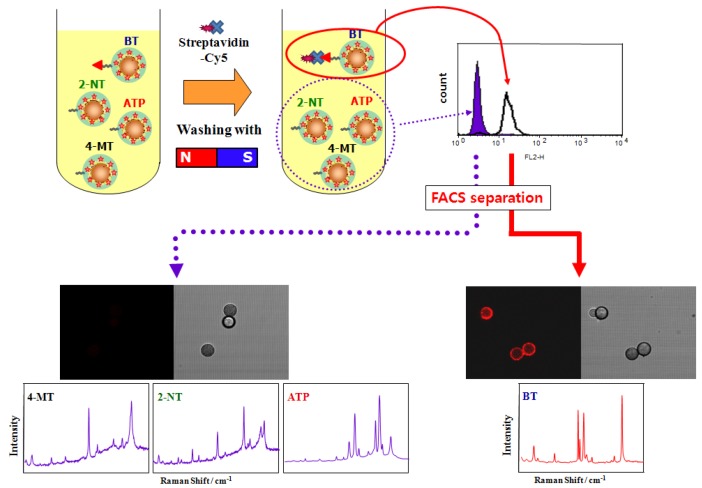
Illustration of applying fluorescence-based protein detection with SERS encoding for HTS system. Fluorescence active streptavidin bound beads were separated using flow cytometry, and analyzed by Raman spectroscopy for recognition of Raman labels and ligand types. (BT: benzenethiol, 4-MT: 4-mercaptotoluene, 2-NT: 2-naphthalenethiol, 4-ATP: 4-aminothiophenol) (reproduced with permission from reference [34]. Copyright 2009, Elsevier B.V.).

The key advantage of this system comes from combination of flow cytometry with optically-encoded beads [45,80]. SERS-encoded beads were applied to conventional fluorescence based flow cytometry to separate target protein bounded beads [34]. In this study, fluorescence-immobilized streptavidin was selectively bound to biotin-immobilized SERS beads among the various ligand-immobilized beads. Then, the target protein-bound beads, which have relatively bright fluorescence, could be separated from the others using flow cytometry, and then the ligands could be recognized by SERS decoding of the beads as shown in Figure 6. The Nolan group has reported SERS-based flow cytometry separation by SERS spectra [81]. They have successfully distinguished four different SERS-encoded beads. 

To take the advantage of the combination of fluorescence-based immunoassays with optically-encoded beads, the choice of fluorescent dye requires special considerations for avoiding spectral overlap. Because fluorescent dyes have narrow excitation wavelengths, they can be selectively excited by laser sources and spectral emission overlap can be avoided. When combining QD-encoded beads and fluorescence-based detection, the overlap can be generally avoided by using emitting spectra, although QDs can be excited by broad wavelengths. Because fluorescence-based coding is based on different emitting wavelengths, combination of fluorescence-based immunoassays with optically-encoded beads could limit coding numbers, and consequently multiplexing ability.

One of main advantages of combining fluorescence-based protein detection with SERS-encoded beads is that the target binding event and the type of ligand can be simultaneously recognized by fluorescence and SERS, respectively, with single laser-line excitation and without interference by coding number.

Fluorescence is quenched by the interaction between metal surfaces and fluorescent dye molecules, and thus, fluorescent dyes are widely used as Raman label compounds to produce resonance Raman signals. However, in the case of using fluorescence and SERS together, fluorescence part is physically separated from silver NPs as SERS substrate, and this prevents quenching of fluorescence.

**Figure 7:**
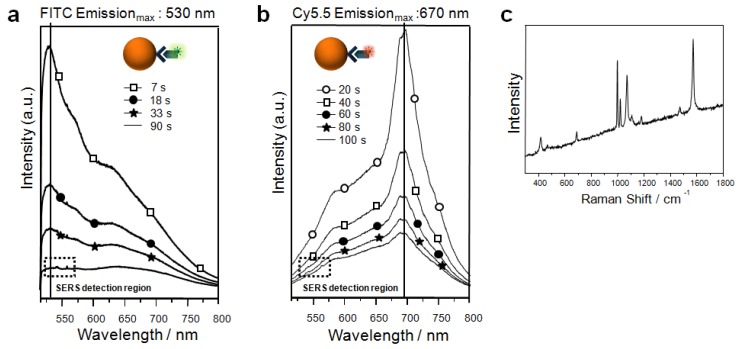
Fluorescence change after photoexcitation by a 514.5-nm laser line on SERS beads incubated with streptavidin-FITC conjugate (**a**) and with streptavidin-Cy5 conjugate (**b**). The corresponding SERS spectrum of (a) is drawn in (**c**) (Reproduced with permission from references 34 and 38. Copyright 2009, Elsevier B.V. and Copyright 2007, American Chemical Society, respectively.).

Another point to be considered is that fluorescence can overlap the SERS spectrum. The best approach is to avoid overlap. For example, when we used FITC (530 nm) or Cy5.5 (670 nm) as fluorescent dyes for target detection in the case of silver-based SERS coding (514-nm laser source), the FITC spectra covered the SERS signal but the Cy5.5 spectra did not, as shown in Figure 7a,b. Thus, the Cy5.5 band at 670 nm did not overlap with Raman signals, and the SERS spectra of 4-BT could be easily recognized, denoting the ligand type, without severe interference from fluorescence background. 

On the other hand, in SERS decoding of fluorescence with SERS beads, broad fluorescence background of FITC at 530 nm almost covered the SERS peaks. This overlap could be avoided by fluorescence photobleaching. Because SERS peaks are not photobleachable, only the intensity of fluorescence was gradually decreased by laser illumination. After about 100 s, fluorescence was almost completely photobleached, and the 4-MT SERS peaks were obtained as shown in Figure 7c. When PDA-based label-free detection methods were applied to SERS-encoded beads, the red PDA-immobilized SERS bioassay is combined with a SERS-encoded bead system, multiplexing of a large number of targets can be accomplished. Moreover, detection of target binding and decoding of coded beads can be beads exhibited fluorescence at 543 nm. At this wavelength, SERS signal could be detected. This illustrates that PDA-based label-free detection methods can be combined with SERS-encoded beads. When a fluorescence-based accomplished by using a single laser source. Therefore, SERS-coded beads can be one of the best candidate methods for bead-based protein detection, and combination of SERS and fluorescence is likely to be useful for multiple protein detection.

## 4. Conclusions and Perspectives

Currently, the analysis of multiple analytes in a single biological sample is required for diagnostic applications. These demands can be met by using multiplex platforms such as planar and bead-based arrays. Bead-based arrays have many advantages in sensitivity, flexibility, and the requirement of small sample volume over planar arrays. In particular, the combination of fluorescence-based detection with optically-encoded beads can provide a robust and efficient approach for setting up multiplexed assays. In this review, we have briefly summarized recent developments in the area of optically-encoded beads based screening of protein molecules. We have also focused on the optically-encoded beads such as multilayer fluorescence beads and SERS-encoded beads which have potential to generate a large number of coding for multiplexing detection. Combination of several strategies like molecular beacon-based techniques or PDA techniques with those beads is also discussed to show the potential for label-free protein.

Even though plenty of success has been, in order for bead-based assays to be more practically achieved in this field, several issues still need to be resolved, including a large number of optical codes, rapid readout method, safety, cost, sensitivity, and ease of use for bioapplications such as multiple protein detection in clinical diagnostics.

(1) With regard to coding materials, unlimited coding number has not been fully accomplished. Fluorescence-based beads can be limited in their number and/or toxicity. Although the coding number of SERS beads has great potential in respect of coding numbers, they have not been completely established. So far, only several to dozens of Raman dyes are widely used, and different signal intensity and signal complexity sometimes limit their practical coding number.
(2) For detection of protein binding, on-beads label-free detection is still at the beginning stage. Development of more smart and practical detection method is necessary to multiplex, fast and sensitive detection.
(3) The great advantages of optically-encoded beads came from well developed decoding and sorting system. So far decoding and sorting with flow cytometry seem to give best performance and can be immediately applicable and promising way.

The combination of fluorescence-based detection and SERS materials could make bead-based assays more attractive in the medical and diagnostic fields. We also expect that the recently developed fluorescence-based label-free method will significantly contribute to the expanded use of bead-based assays.
